# The role of post-mortem investigations in deaths due to fermenting grape gas: A case series of four fatalities

**DOI:** 10.1016/j.fsisyn.2024.100561

**Published:** 2024-11-12

**Authors:** Matteo Antonio Sacco, Saverio Gualtieri, Loris Rivalta, Caterina Nesci, Raffaele La Russa, Cristina Juan, Pietrantonio Ricci, Isabella Aquila

**Affiliations:** Institute of Legal Medicine, Department of Medical and Surgical Sciences, “Magna Graecia” University, 88100, Catanzaro, Italy; ASP Catanzaro, 88100, Catanzaro, Italy; Department of Clinical Medicine, Public Health, Life Sciences, Environmental Sciences, University of L'Aquila, 67100, L'Aquila, Italy; Laboratory of Food Chemistry and Toxicology, Faculty of Pharmacy and Food Science, Av. Vicent Andrés Estellés S/n, 46100, Burjassot, University of Valencia, València, Spain; Institute of Legal Medicine, Department of Medical and Surgical Sciences, “Magna Graecia” University, 88100, Catanzaro, Italy

**Keywords:** Forensic sciences, Carbon dioxide, Carbaminohemoglobin

## Abstract

Carbon dioxide (CO_2_) is an endogenous molecule produced by cellular respiration and therefore present in our body and in the atmosphere. It is known that, at high levels, CO_2_ causes fatal toxic effects. The production of CO_2_ can prove particularly dangerous in closed and unventilated environments. Although some cases of death due to CO_2_ are described in forensic literature, today this diagnosis of CO_2_ intoxication on cadaver is still a challenge as it is a volatile endogenous molecule and strongly increased after death due to anoxia. In this paper, we describe six cases of simultaneous CO_2_ intoxications which was caused by a fermentation of grape must in a closed environment. Four people died while the other two were rescued. Scene investigation with measurement of environmental gas levels using sensors was performed. Autopsies were carried out with toxicological investigations and histopathological analyses. Finally, a review of the scientific literature on deaths related to fermenting grape gas was carried out. The results demonstrated toxic levels of CO_2_ at the scene. The corpses showed initial transformative phenomena such as skin discoloration and slippage, while toxicological investigations demonstrated a peak of HbCO_2_ (carbaminohemoglobin) with alcohol positivity in the blood of the deceased subjects. Histopathological investigations showed must yeasts (*Saccharomyces*) in the pulmonary alveoli. The paper reports the procedures performed proposing the introduction of new experimental investigations for the post-mortem diagnosis of CO_2_ intoxication with a forensic diagnostic protocol.

## 1 Introduction

**Fig. 1:**
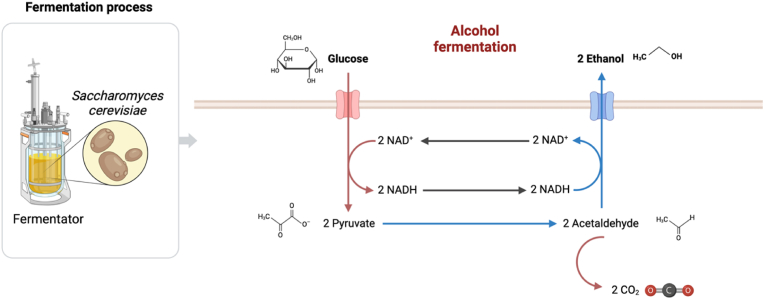
Chemical structure for the process of must fermentation (created with Biorender.com).

Alcoholic fermentation is a chemical process carried out during winemaking, after pressing the grape must. Fermentation lasts up to 15 days and is carried out by yeasts of the *Saccharomyces cerevisiae* species [1]. In an initial phase, yeasts use an aerobic respiration mechanism by exploiting oxygen to transform sugars into carbon dioxide (CO_2_) and water [2]. The second phase is that of the actual fermentation which occurs when the aerobic metabolism becomes anaerobic due to the exhaustion of oxygen in the must. In anaerobic conditions, pyruvic acid, produced by the glycolysis process, is converted by yeasts into ethyl alcohol (50 %) and carbon dioxide (45 %) [[3], [4], [5], [6], [7]] (Fig. 1).

CO_2_ is an odorless and colorless gas. These physical characteristics make the identification of the molecule at toxic levels very complex and its high density facilitates its saturation in a closed environment with lethal effects. In forensic literature, death from must exhalations is a rare event. According to literature, the reported cases often occur in an accidental manner due to closed and easily saturated environments or in a suicidal manner through plastic bag suffocation. Investigations performed generally include autopsy and standard toxicological investigations. Due to the limitations described, the analysis of these cases is today mostly limited to the collection of circumstantial data found at the scene. Post-mortem identification of CO_2_ intoxication is important for reconstructing the event but the analysis is limited due to the volatility of CO_2_ which make it complex to prove exposure on biological fluids.

A literature review was carried out in the field of deaths from CO_2_ and must poisoning. The search was carried out on the PubMed NCBI and SCOPUS search engines following the PRISMA guidelines. The following key words *carbon dioxide and death and intoxication and forensic* were used. Only full-papers in English were analyzed. Similar works that emerged from the search engine suggestions were also analyzed. Papers included all types of CO2 poisonings except those relating to inhalation of volcanic gases. Non-English language works were excluded. The results obtained were compared.

The literature review identified a total of 45 papers published between 1994 and 2013. Once duplicates had been eliminated, 15 papers were selected based on the title and abstract according to the inclusion criteria. After reading the full papers, the literature review therefore identified a total of 9 scientific papers with descriptions of forensic cases (Table 1). A paper was extracted from collateral bibliography [8]. Within the 10 papers selected, a total of 16 cases of CO_2_ poisoning were reported. In 5 papers the manner of death was accidental; in 4 papers suicides were described, in 1 paper a multiple homicide was reported. In 3 papers, the toxicological findings were positive for alcohol. In the other papers toxicological analysis were negative or inconclusive.

**Table 1:** Review results of forensic papers about CO_2_ intoxication.

Authors and year	Number of cases (1 case = 1 person)	Manner of death	Environment	Autopsy findings	Toxicological findings	Other forensic results
Nakamura et al., 2023 [9]	1	Accidental	Using dry ice in a burial room	Visceral congestion	Positive for alcohol	The reconstruction of the experimental model proved that after 9 hours toxic levels of CO_2_ were reached
Wen et al., 2022 [10]	2	Accidental	Fire suppressant storage room	Purplish-reddish skin discolorations; Spotted subcutaneous hemorrhages; Pulmonary congestion with edema	Negative, with non-toxic levels of CO	Antemortem blood gas analysis showed severe acute hypercapnia accompanied by respiratory acidosis.
Murakami et al., 2018 [11]	1	Suicide	Plastic bag suffocation containing citric acid solution and baking soda	Dark-reddish skin discolorations; Numerous petechial hemorrhages on the upper body and heart; Congestion; cerebral haemorrage	Negative	**-**
Handlos et al., 2018 [12]	2	Accidental	Silage Pit	Maceration of the skin; pinkish fluid in the airways; pleural petechiae	Negative	**-**
Sautter et al., 2014 [13]	4	Homicide-suicide	CO(2)-fertilizer for aquarium plants using a mask for the victims and a plastic bag for the killer	Presence of petechiae and infarct of the left ventricular anterolateral papillary muscle on the killer Putrefaction	Positive for alcohol on the killer.	DNA of the victims was found on the mask analyzed
Rupp et al., 2013 [14]	2	Suicide	Use of dry ice in case 1; Closure in a cooler containing CO_2_ in case 2	Putrefaction in the first case; Haemorragic pulmonary edema, and cerebral edema in the second case	Positive for alcohol in both cases	**-**
Kettner et al., 2013 [15]	1	Accidental	Fermentation tank	bruises, head hematoma, subconjunctival petechiae, myocardial hemorrhage, and edema of the lungs	Negative, including CO	High CO_2_ content on spectrometric analysis of the lung
La Harpe et al., 2013 [8]	1	Accidental	Fermentation tank	petechiae, cyanosis, multiple skin bruises, cerebral edema, congestion	Negative, including CO	–
Srisont et al., 2009 [16]	1	–	Closed plastic container	Moderate congestion, mild cerebral edema	Negative	
Dedouit et al., 2008 [17]	1	Suicide	Room for the packaging of fruits and vegetable	–	Negative, including CO	The partial arterial CO_2_ blood level was 204 mmHg

CO_2_ is a natural gas also present in the human organism with an alveolar concentration of 5 %(8; 10–17). At high levels, however, this molecule can cause significant toxicity with relevant clinical effects on the central nervous, cardiovascular and respiratory systems. In fact, carbon dioxide stimulates circulation, breathing and heart rate even at slightly high pCO_2_ values (>10 vol%) causing tachypnea and tachyarrhythmia [13]. Neurological effects have been described starting from pCO_2_ values equal to a volume of 10 %. They include headache, anxiety, and paralyzing effects with altered states of consciousness and reduced excitability. Levels between 10 and 20 % can cause adverse neurological effects such as epileptic seizures and coma.

Higher effects, with a pCO_2_ between 20 and 30 % can cause fatal effects. Murakami et al. described how such high CO_2_ concentrations can cause loss of consciousness and respiratory arrest within a minute [11]. From a respiratory point of view, the authors described how the increase in CO2 levels leads to a stimulation of the inspiratory centers generating a very strong sensation of asphyxia. From a cardiovascular point of view, CO_2_ causes an acceleration of the heart rate. This tachycardia, caused by the stimulation of the sympathetic system, generates a compensatory peripheral vasodilation with entry, in case of exposure to toxic levels of CO_2_, of an even higher proportion of the molecule at the brain and cellular level, enhancing its effects. From a metabolic point of view, this results in a significant lowering of pH in the cell, causing a severe respiratory acidosis at a systemic level. It has been described that already at 75 mmHg, CO2 determines the first neurogenic effects and at 150 mmHg it can cause a fatal cardiac arrhythmia [18].

The post-mortem identification of CO_2_ levels in a corpse is difficult, both due to the volatile characteristics of the gas and because after death the O_2_ levels drop and the CO_2_ levels rise [12]. Therefore, since it is a molecule naturally present in the organism, it is difficult to discriminate and measure overexposure to CO_2_ with objective data.

In the previous reports, in all cases reported in forensic literature, there is a real difficulty in post-mortem toxicological investigations on this molecule. In one paper alone, Kettner et al. described a high concentration of CO_2_ identified by quadrupole-mass-spectrometry on a lung subjected to vacuum in a case of CO_2_ intoxication [15]. In other cases, the diagnosis was made through scene investigations, standard toxicology tests and autopsy (8; 16–17). We emphasize how the review of the literature has described, among the most frequent autopsy findings, an earlier putrefaction, presence of petechiae, infiltration of the skullcap, skin discoloration, pulmonary congestion with edema. In this paper, we describe how the literature results were also confirmed in the reported cases.

The aim of this work is to illustrate an innovative experimental analysis approach through the use of histopathogical, toxicological (including HbCo_2_ determination), and autopsy data in must related incidents. The investigations carried out are relevant in the international forensic panorama as, to our knowledge, this represents the case of must intoxication with the highest number of victims described in the literature review presented.

### 1.1 Case presentation

In a southern Italy city, four winemakers started winemaking procedures since the morning. One of the operators, involved in transferring the must into the barrels, went down into a tank when he began to show signs of losing consciousness and collapsed in the tank with his body immersed in the must. Meanwhile, a second man entered the tank but immediately left due to the air becoming unbreathable. Subsequently, a third and fourth man went down into the tank in an attempt to save the first but they also lost consciousness and collapsed with their bodies and heads entirely immersed in the must due to the rise in its level. So, the first, third and fourth men died. Subsequently, a fifth man and a woman went down into the tank in a last-ditch attempt to save everyone. The fifth man died. The woman was recovered and placed in safety after cardiopulmonary resuscitation maneuvers following a cardiorespiratory arrest and she was subsequently transported in conditions of severe respiratory failure with hypoxemia, by air ambulance. The surviving second man did not require admission to hospital. In total, the “domino-effect” incident resulted in the death of four men and the acute intoxication of a man and a woman who were saved (Table 2) [19].

**Table 2:** Cases analyzed.

Cases	Sex	Age	Post-mortem interval	Cause of death	Other drugs detected	Death scenarios
Case 1 (fourth man)	M	63	53 hours	Asphyxia	Alcohol	Found inside the tank
Case 2 (third man)	M	45	53 hours	Asphyxia	Alcohol	Found inside the tank
Case 3 (first man)	M	69	53 hours	Asphyxia	Alcohol	Found inside the tank
Case 4 (fifth man)	M	51	53 hours	Asphyxia	Alcohol	Found outside the tank

## 2 Material and methods

### 2.1 Scene investigation and autopsy

The investigators carried out a scene inspection with the help of the fire brigade, using special respirators to guarantee the operators safety. Subsequently, a qualitative and quantitative measurement of the ambient gas and fumes was carried out on site using a special atmospheric gas level sensor. The investigators also proceeded to evaluate the environments safety and to collect testimonial information from people present. The scene analysis demonstrated that the tank was inside a closed environment below the walking level. The tank had no window or possibility of air exchange and the only way out was a staircase. Environmental analyzes through the sensors used on the scene reported toxic levels of CO_2_ saturation with the presence of sulfuric acid.

**Fig. 2:**
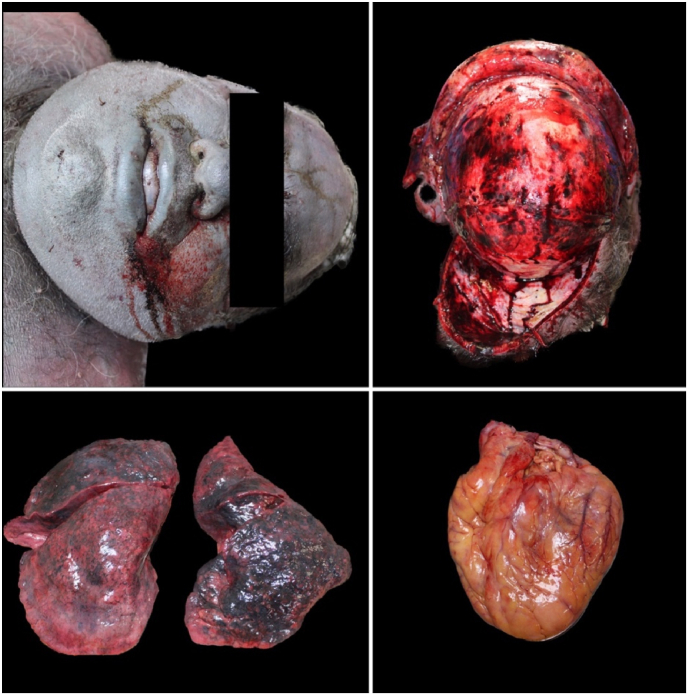
Autopsy gross findings in case 1.

**Fig. 3:**
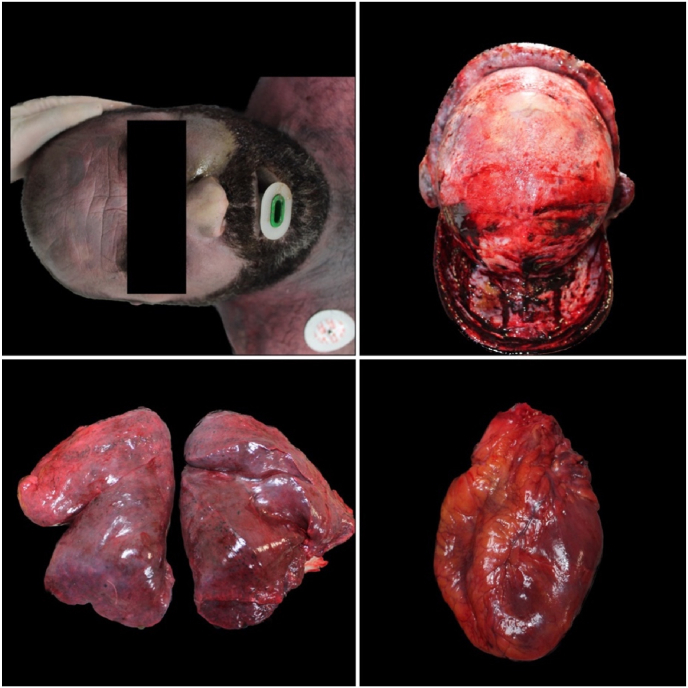
Autopsy gross findings in case 2.

**Fig. 4:**
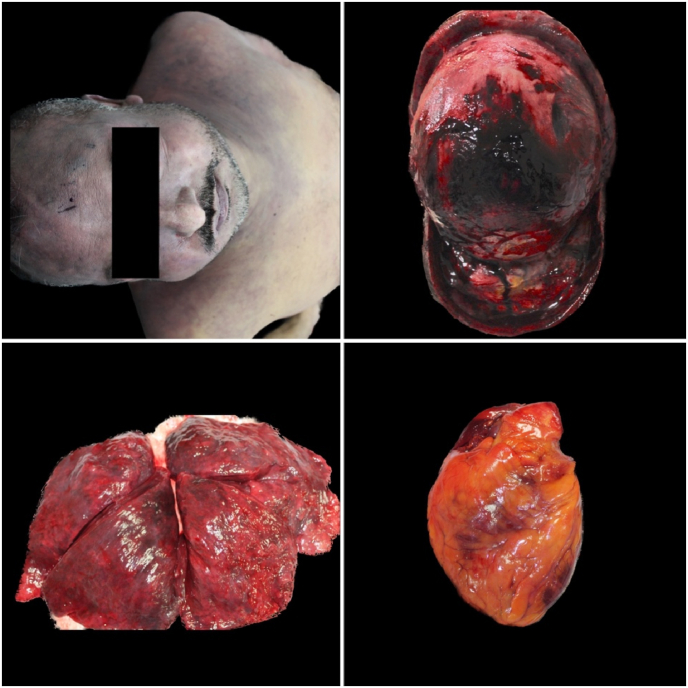
Autopsy gross findings in case 3.

**Fig. 5:**
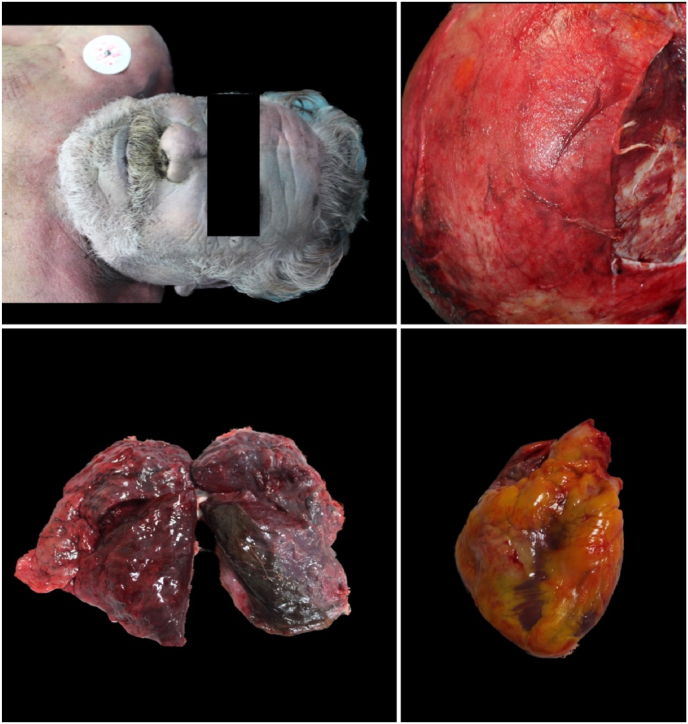
Autopsy gross findings in case 4.

An external examination with autopsy was then carried out on all four cadavers. The clothing of all four subjects were damp with and intense smell of alcohol. Sampling of biological fluids and gross analysis of all organs was completed. Analysis of the glottis-trachea-lung block was performed. Samples of all organs were taken for histopathological investigations. All specimens collected were fixed in 10 % formalin and subsequently subjected to processing, embedding and staining with hematoxylin-eosin [20]. The autopsy showed initial putrefactive phenomena in all cases with signs of skin maceration. The cadavers externally presented intense violaceous hypostasis, resolved rigidity, opaque corneas. In particular, the forensic pathologists found intense subconjunctival hemorrhage, and nonexternal traumatic injuries were found. At autopsy, however, an intense hemorrhagic infiltrate on parieto-occipital region was evident on the skull. The corpses therefore all showed signs of asphyxiation, including petechiae, dark and fluid blood, cyanosis (Fig. 2, Fig. 3, Fig. 4, Fig. 5). All brains appeared severely congested. The lungs showed significant emphysema with pulmonary hyperexpansion, subpleural petechiae and perihilar lymph node reactivity, and pulmonary edema on section. All aortas showed an intense red color and all organs showed strong visceral congestion. The stomaches contained pinkish liquid contents.

### 2.2 Toxicological investigations

Toxicological screening analysis for alcohol and drugs was performed on peripheral blood and vitreous humor samples from the four decedents. No toxicological investigations were carried out by the physicians on the two surviving subjects. A variety of analytical techniques were employed and these are described in Table 3.

**Table 3:** List of chemicals and instruments for each analytical technique. With the exception of distilled water produced in-house, all chemicals were purchased from chemical supply company such as “Thermo Scientific Chemicals (Germany)” at technical grade or better and were used as received without further purification.

Analytical techniques	Chemicals	Instruments
Screening test	Acetonitrile, phosphate buffer	Werfen ILAB650 Clinical Chemistry System (software ILAB650 rev.02 09/08)
Alcohol screening	trichloroacetic acid, phosphate buffer, NaOH	Werfen ILAB650 Clinical Chemistry System (software ILAB650 rev.02 09/08)
Alcohol confirmation	butanol	Agilent Carboxen/PDMS Agilent 7890- 5975C Gas chromatography combined with mass spectrometry (GC-MS)
Determination of carboxyhemoglobin	Sodium dithionite, phosphate buffer, distilled water	Beckman DU530 UV–Vis Spectrophotometer
Determination of carbaminohemoglobin	–	Beckman DU530 UV–Vis Spectrophotometer)

The rationale for the screening was to evaluate the presence of any exogenous substances taken by the subjects through a first level investigation. For the screening test, the blood was treated with acetonitrile and subsequently centrifuged (Centric MF48/-R) for 5 minutes at 3000 rpm. The liquid part obtained was then dried at a temperature of 70–80 °C and then treated with 0,1M pH 6 made in-house phosphate buffer and analyzed with ILAB650 Instrumentation with immunoenzymatic method [21]. The samples were loaded directly into tubes in a volume of 30 μL together with the reagents and the results were subsequently read by the appropriate software (ILAB650 rev.02 09/08), according to in-house procedure. For the extraction of alcohol, the procedure involved the use of trichloroacetic acid and centrifugation for 5 minutes at 3000 rpm. The liquid part was then returned to a separate test tube with the addition of 1 M pH 6 made in-house phosphate buffer and concentrated NaOH and analysis with ILAB650 Instrumentation with immunoenzymatic method [18]. Confirmation of the blood alcohol level was carried out by extraction on 10 μL of blood, placed in a 10 ml vial and closed with a metal ring with the addition of 10 μL of butanol and using a Carboxen/Polydimethylsiloxane (PDMS) solid phase microextraction (SPME) fiber assembly was used as standard. The quantitative determination was carried out in SIM (Selected Ion Monitoring) using SPME with gas chromatography combined with mass spectrometry (GC-MS) [[21], [22], [23], [24]]. In all four cases, alcohol was detected with levels between 1.37 and 2.23 g/L.

The spectrophotometric determination of carboxyhemoglobin was completed using whole blood samples. After treatment of the blood with distilled water and after centrifugation (5 minutes at 3000 rpm), a 200 μL sample was taken from the hemolysates obtained and added to a previously prepared 2,8 ml sodium dithionite solution (prepared dissolving 22.5 mg of sodium dithionite in 15 ml of 0.1 pH 6 made in-house phosphate buffer) and the sample was then subjected to spectrophotometric analysis [17]. Finally, the blood samples were subjected to in-house spectrometric scanning (Headspace Solid-Phase Micro-Extraction (HSPME) with dynamic technique) for the search for carbaminohemoglobin (HbCO_2_), i.e. an analysis of the amount of hemoglobin with CO_2_ binding to the central iron and the terminal lysine. The samples were compared with blood from control cases subjected to the same analysis.

## 3 Results and discussion

### 3.1 Histopathological findings

**Fig. 6:**
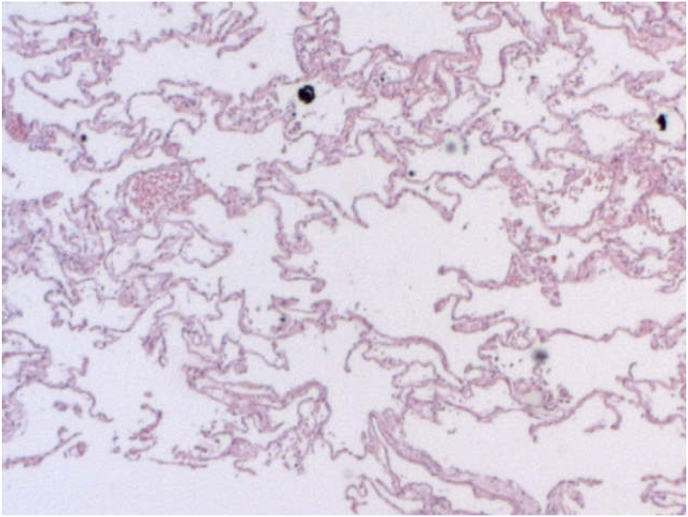
Areas of intraalveolar atelectasis characterized by collapse of alveoli with loss of volume.

**Fig. 7:**
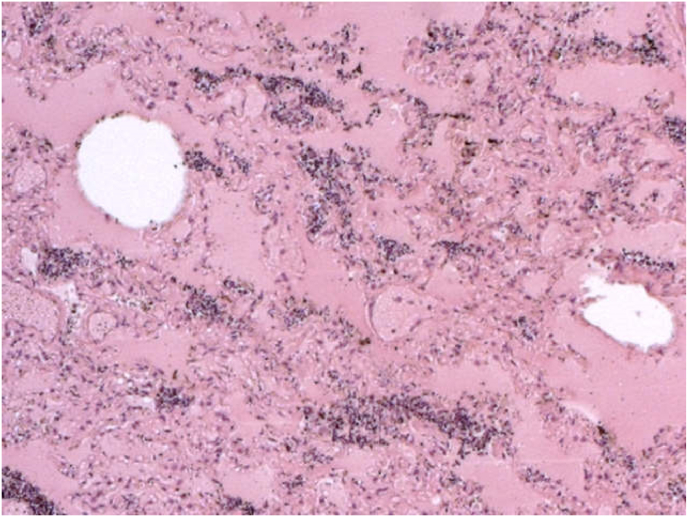
Rounded or oval elements, sometimes pseudonucleolated, arranged in groups in the interstitial and alveolar spaces.

**Fig. 8:**
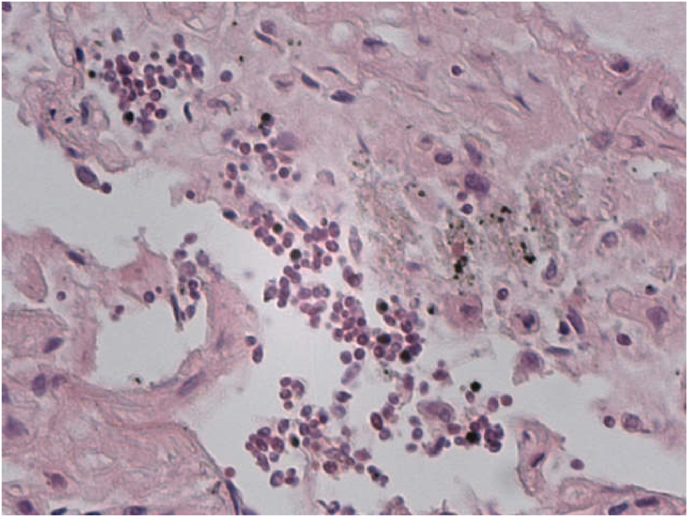
Detail of Saccharomyces in lungs recognizable as group of cells with globose and elliptical shape with a diameter of approximately 5–10μm.

Histopathological analysis showed marked vascular congestion in the lungs, areas of intraalveolar edema and atelectasis alternating with others of emphysema. The microscopic examination identified the presence of rounded or oval elements, sometimes pseudonucleolated, arranged in groups in the interstitial and alveolar spaces, morphologically compatible with elements of a fungal nature (must yeasts) such as *Saccharomyces* (Fig. 6, Fig. 7, Fig. 8). The examination of the other organs confirmed plurivisceral congestion in the other organs, with a state of moderate autolysis.

### 3.2 Toxicological findings

Toxicological analysis showed in all four cases the presence of alcohol in the blood with levels between 1.37 g/L and 2.23 g/L (Table 4). Tests for all other drugs were negative. The concentration of CO was instead between 6.5 % and 9.9 % in the cases analyzed and therefore at a non-toxic level (toxicity range >20 %) [15]. The levels of HbCO_2_ in all four cases showed a peak (30–38 %), comparable to the CO_2_ results described by Kettner et al., as an indication of prolonged exposure to CO_2_ [15].

**Table 4:** Toxicological results on examined cases.

Cases	Alcohol blood levels	CO blood levels	HbCO_2_ blood levels
Case 1	1,37 g/L	6,5 %	30 %
Case 2	1,84 g/L	9,9 %	32 %
Case 3	2,23 g/L	9,9 %	38 %
Case 4	1,41 g/L	9,7 %	31 %

Death occurred due to asphyxia with confinement for lack of oxygen and overproduction of CO_2_ in a stale atmosphere caused by the fermenting must. In our cases we report, to the best of our knowledge, for the first time in the literature the post-mortem identification of a HbCO_2_ peak in the blood of the four subjects as a potential index of prolonged exposure for all CO_2_ poisoning cases. We also highlight the presence of ethanol found in circulation at the time of death in all subjects, probably attributable to inhalation or concomitant ingestion of alcohol as well as rapid putrefaction despite regular storage in a cold room, probably related to the possible accelerating role on putrefaction of the inhaled gas. We also evidence the fundamental role that histopathological investigations play in cases of CO_2_ intoxication caused by fermenting must, considering that the microscopic analyzes identified the Saccaromyces fungus in the lungs of all four subjects as an essential yeast for fermentation. The presence of Saccharomyces in alveoli proved that the individuals suffered a phase of inhalation of wine must in lungs.

We therefore propose, in cases of suspected CO_2_ intoxication, the following forensic investigation protocol.

**Fig. 9:**
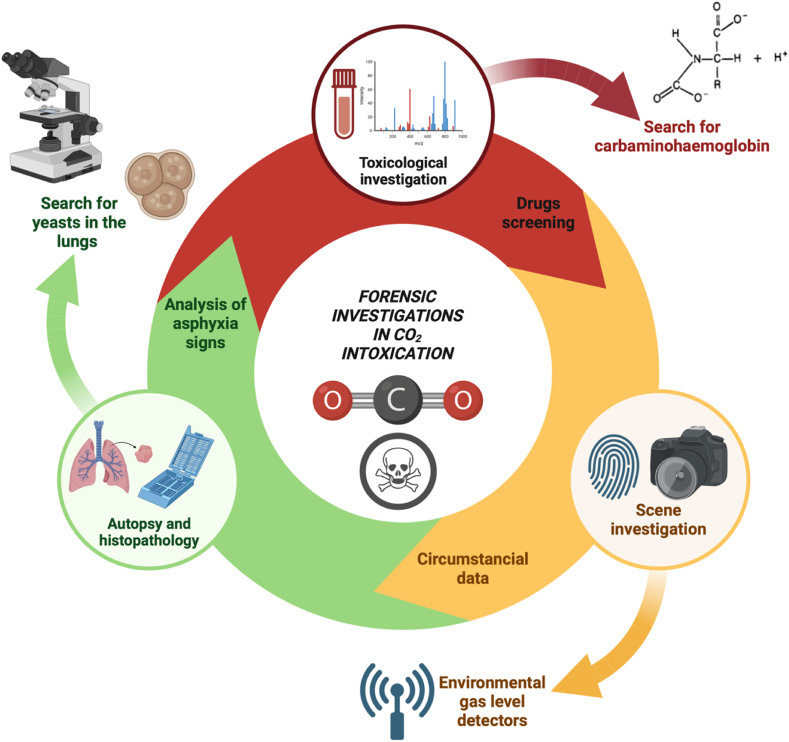
Forensic investigation protocols in CO_2_ intoxication cases (created with Biorender.com).

- Analysis of the scene with environmental gas level detectors;
- Autopsy examination with evaluation of putrefaction, analysis of signs of asphyxia, examination of the skullcap;
- Toxicological investigations on blood with research for HbCO_2_ using spectrometry;
- Histopathological examination with search for yeasts in the lungs (in case of must fermentation);
- Evaluation of security systems and deficiencies at the scene;
- Any advanced diagnostic investigations such as spectrometry on samples of lung tissue or other organs, if available (Fig. 9).

## 4 Conclusions

The cases illuminate the need for maximum caution during winemaking operations, in particular when carried out in closed, non-ventilated and unsafe environments. In these cases, we propose a multidisciplinary investigative protocol that include autopsy and histopathological and toxicological findings, noting a potential role of HbCO_2_ in diagnostics. The value of this research is to overcome the limits to prove death to CO_2_ intoxication, due to its volatility, offering concrete post-mortem evidences. Further studies will be necessary in the future to confirm the role of this molecule as a potential key for the diagnosis of CO_2_ intoxication. Such cases require suitable forensic investigations to assess responsibility for the lack of safety and ventilation systems.

## CRediT authorship contribution statement

**Matteo Antonio Sacco:** Writing – original draft, Conceptualization. **Saverio Gualtieri:** Writing – original draft. **Loris Rivalta:** Methodology. **Caterina Nesci:** Methodology. **Raffaele La Russa:** Formal analysis, Data curation. **Cristina Juan:** Formal analysis, Data curation. **Pietrantonio Ricci:** Investigation. **Isabella Aquila:** Writing – original draft, Supervision, Conceptualization.

## Declaration of competing interest

The authors declare that they have no known competing financial interests or personal relationships that could have appeared to influence the work reported in this paper.
